# Oral Administration of Caffeine Exacerbates Cisplatin-Induced Hearing Loss

**DOI:** 10.1038/s41598-019-45964-9

**Published:** 2019-07-02

**Authors:** Sandeep Sheth, Kelly Sheehan, Asmita Dhukhwa, Raheem F. H. Al Aameri, Chaitanya Mamillapalli, Debashree Mukherjea, Leonard P. Rybak, Vickram Ramkumar

**Affiliations:** 1 Department of Pharmacology, Southern Illinois University School of Medicine, Springfield, IL United States; 2 Department of Otolaryngology, Southern Illinois University School of Medicine, Springfield, IL United States; 3 Department of Internal Medicine (Division of Endocrinology), Southern Illinois University School of Medicine, Springfield, IL United States

**Keywords:** Cochlea, Adverse effects

## Abstract

Adenosine A_1_ receptors (A_1_AR) are well characterized for their role in cytoprotection. Previous studies have demonstrated the presence of these receptors in the cochlea where their activation were shown to suppress cisplatin-induced inflammatory response and the resulting ototoxicity. Inhibition of A_1_AR by caffeine, a widely consumed psychoactive substance, could antagonize the endogenous protective role of these receptors in cochlea and potentiate cisplatin-induced hearing loss. This hypothesis was tested in a rat model of cisplatin ototoxicity following oral administration of caffeine. We report here that single-dose administration of caffeine exacerbates cisplatin-induced hearing loss without increasing the damage to outer hair cells (OHCs), but increased synaptopathy and inflammation in the cochlea. These effects of caffeine were mediated by its blockade of A_1_AR, as co-administration of *R*-PIA, an A_1_AR agonist, reversed the detrimental actions of caffeine and cisplatin on hearing loss. Multiple doses of caffeine exacerbated cisplatin ototoxicity which was associated with damage to OHCs and cochlear synaptopathy. These findings highlight a possible drug-drug interaction between caffeine and cisplatin for ototoxicity and suggest that caffeine consumption should be cautioned in cancer patients treated with a chemotherapeutic regimen containing cisplatin.

## Introduction

Cisplatin is a critical component of chemotherapeutic regimen used for the treatment of several kinds of cancers. Unfortunately, cisplatin produces a debilitating side effect of permanent hearing loss which severely affects the quality of life of cancer patients. Cisplatin is known to target numerous cell types in the basal and middle turn of the cochlea, including outer hair cells (OHCs)^1,2^, supporting cells^3^, marginal cells of stria vascularis (SVA)^4,5^, spiral ligament (SL)^6^ and spiral ganglion neurons (SGN)^7–9^, but does not seem to affect the inner hair cells (IHCs)^10,11^. One of the important molecular mechanisms responsible for hearing loss induced by cisplatin is generation of reactive oxygen species by weakening cochlear antioxidant defense system^2^. Recent studies from our lab suggest that the increased ROS generation initiates an inflammatory process by activating signal transducer and activator of transcription1 (STAT1)^10,12,13^. STAT1 is a transcription factor that regulates the expression of inflammatory genes such as tumor necrosis factor-α (TNF-α)^14^, inducible nitric oxide synthase (iNOS)^15^ and cyclooxygenase-2 (COX-2)^16^.

Adenosine receptors are G protein-coupled receptors which mediate the physiological effects of the ubiquitously present signaling molecule, adenosine^17^. Although all four adenosine receptors (AR), namely A_1_, A_2A_, A_2B_ and A_3_, are expressed in the cochlea^18^, activation of A_1_AR specifically has been found to protect from cisplatin-induced hearing loss^10,19–21^. We have tested the effectiveness of A_1_AR specific agonist, N^6^-*R*-phenylisopropyladenosine (*R*-PIA), in a rat model of cisplatin ototoxicity and showed that this agent protected against hearing loss when administered via the trans-tympanic route^10^. We demonstrated that A_1_AR-mediated otoprotection involved suppression of ROS-dependent inflammatory response in the cochlea through inhibition of the STAT1 transcription factor^10^.

Since activation of A_1_AR protects against cisplatin ototoxicity, any agent that blocks A_1_AR signaling could exacerbate cisplatin-induced hearing loss. Caffeine (1,3,7-trimethylxanthine) is one of the most widely consumed dietary psychoactive stimulant throughout the world^22^. High amounts of caffeine is present in coffee, carbonated drinks and energy drinks and the average daily intake of caffeine in adults in the US is ~280 mg/day^22^. Pharmacologically, caffeine acts as CNS stimulant by non-selectively binding to and inhibiting ARs^23^. This A_1_AR antagonistic property of caffeine in the cochlea could aggravate cisplatin-induced hearing loss. This hypothesis is supported by a recent study which showed that caffeine has detrimental effect on hearing recovery after a single event of acoustic trauma^24^. In this study, we test whether oral caffeine consumption would exacerbate cisplatin ototoxicity by inhibiting the protective action of the A_1_AR in the cochlea. A negative interaction would infer that caffeine should be contraindicated in cancer patients who are on chemotherapeutic regimen containing cisplatin.

## Results

### Oral administration of caffeine exacerbates cisplatin-induced hearing loss in rats

**Figure 1:**
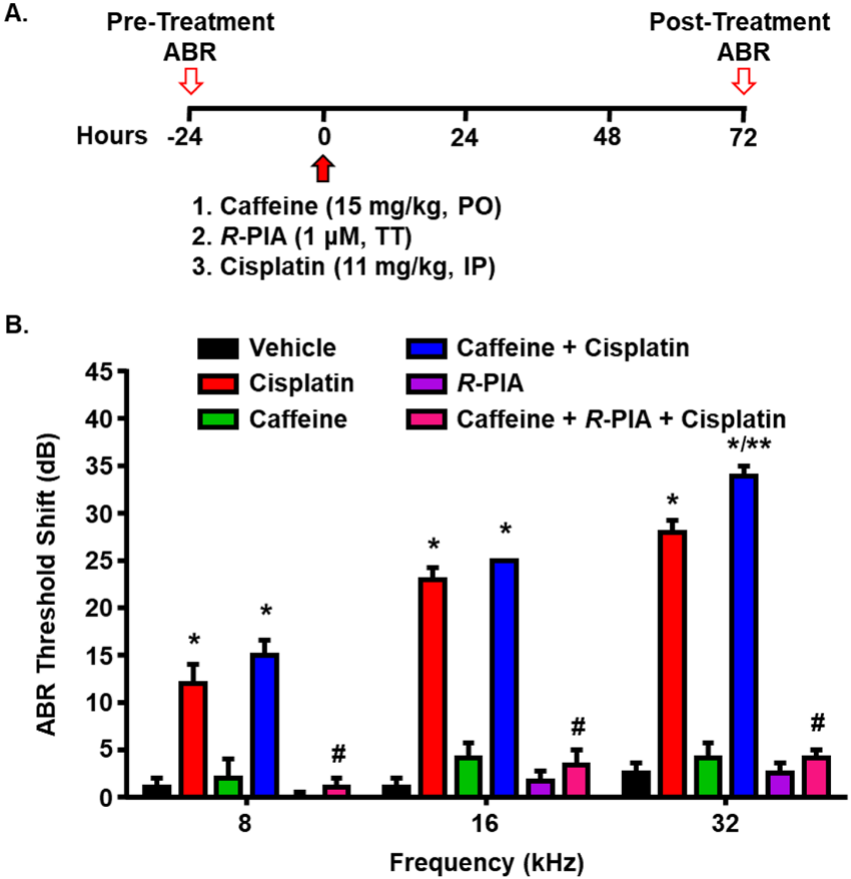
Oral administration of caffeine aggravates cisplatin-induced hearing loss. (**A**) A schematic of the experimental protocol that depicts the dosage and routes of administration of different drugs. Wistar rats were divided into six groups and treated with either oral caffeine (15 mg/kg), followed by trans-tympanic delivery of vehicle or *R*-PIA (1 μM) in both ears. This was followed by intraperitoneal administration of vehicle or cisplatin (11 mg/kg) 30 min later. Pre-treatment ABRs were measured just before the drugs were administered and post-treatment ABRs was determined 72 h following cisplatin. PO: Per os (oral); TT: Trans-tympanic; IP: Intraperitoneal. (**B**) ABR thresholds were recorded in Wistar rats treated with cisplatin (11 mg/kg, IP) after oral administration of caffeine (15 mg/kg) or caffeine + *R*-PIA (1 µM, TT). Post-treatment ABRs assessed 3 days later showed significant elevations in thresholds in cisplatin and caffeine + cisplatin groups at 8 and 16 kHz frequencies. At 32 kHz, caffeine co-administration produced hearing loss which was significantly greater than cisplatin alone. Trans-tympanic delivery of *R*-PIA attenuated the increase in ABR thresholds produced by caffeine + cisplatin at all the frequencies tested. Data indicate mean ± SEM of five rats. Asterisks, *p < 0.05 *vs*. vehicle, **p < 0.05 *vs*. cisplatin and ^#^*p* < 0.05 *vs*. caffeine + cisplatin (one-way ANOVA).

Immunohistochemical studies from our laboratory have clearly shown the expression of A_1_ARs in the inner and outer hair cells of the rat cochlea^10^. Furthermore, activation of these receptors through round window application of *R*-phenylisopropyladenosine (*R*-PIA), a selective A_1_AR agonist, reduced cisplatin-induced hearing loss and hair cell damage in chinchilla cochlea^21^. We saw a similar effect of the administration of *R*-PIA by the trans-tympanic route in rats^10^. These studies support the otoprotective role of cochlear A_1_AR against cisplatin-induced hearing loss. We hypothesized that administration of caffeine, a non-selective AR antagonist, could counteract this otoprotection afforded by A_1_AR and exacerbate cisplatin-induced hearing loss. To test this, male Wistar rats were divided in six groups and were administered oral caffeine (15 mg/kg), followed by trans-tympanic delivery of *R*-PIA (1 µM) in both ears. The rats were then treated with intraperitoneal injection of cisplatin (11 mg/kg) 30 mins later. Appropriate vehicle was administered in these animals that did not receive the drugs (Fig. 1A). Administration of vehicle, caffeine and *R*-PIA alone produced minimal change in ABR threshold when compared to the pre-treatment ABR measurements (Fig. 1B). Cisplatin increased the ABR threshold by 12.0 ± 2.0, 23.0 ± 1.2 and 28.0 ± 1.2 dB at 8, 16 and 32 kHz, respectively. Co-administration of caffeine with cisplatin also produced a significant shift in ABR threshold at 8 (15.0 ± 1.6 dB) and 16 (25 dB) kHz frequencies as compared to vehicle, but this ABR shift was statistically similar to that produced by cisplatin. At 32 kHz however, caffeine co-administration with cisplatin produced hearing loss which was significantly greater than cisplatin alone. The ABR threshold shift at 32 kHz was 34.0 ± 1.0 by caffeine + cisplatin treatment group. The hearing loss produced by caffeine + cisplatin was completely blocked by trans-tympanic administration of *R*-PIA at all the frequencies tested. The ABR threshold shift in animals treated with caffeine + *R*-PIA + cisplatin was 1.0 ± 1.0, 3.3 ± 1.7 and 4.2 ± 0.8 at 8, 16 and 32 kHz, respectively. These findings suggest that single oral administration of caffeine exacerbates cisplatin-induced hearing loss, at least at 32 kHz frequency. Moreover, *R*-PIA attenuated the hearing loss produced by caffeine + cisplatin, implicating A_1_AR as the target of caffeine.

### Caffeine consumption does not aggravate cisplatin-induced OHC loss

**Figure 2:**
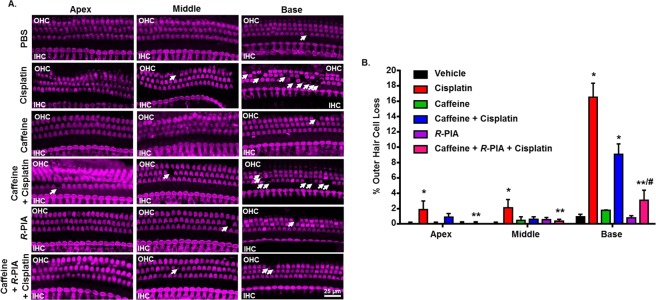
Caffeine consumption does not intensify cisplatin-induced loss of OHCs. (**A**) Cochleae isolated from treated animals were decalcified and micro-dissected into apical, middle and basal turns, which corresponds to 8, 16 and 32 kHz frequency regions, respectively. These whole mount sections were then stained with hair cell marker, Myosin VIIa (magenta). Representative images show significant OHC damage (white arrows) by cisplatin in apical, middle and basal turns. Caffeine co-administration did not exacerbate cisplatin-induced OHC damage. Administration of *R*-PIA reversed the loss of OHC caused by cisplatin. Scale bar represents 25 μm. (**B**) Bar graph from the data presented in (**A**) shows percentage of OHC loss in the apical, middle and basal turns. Counting is based on 150 OHCs per cochlea. Data indicates mean ± SEM (n ≥ 3). Asterisks, *p < 0.05 *vs*. vehicle, **p < 0.05 *vs*. cisplatin and ^#^*p* < 0.05 *vs*. caffeine + cisplatin (one-way ANOVA).

Cisplatin-induced hearing loss was associated with the loss of OHCs in the apical, middle and basal turns of the cochlea, which correspond to 8, 16 and 32 kHz frequency regions, respectively (Fig. 2A,B). Manual counting of OHCs in whole-mount preparations in cisplatin-treated group indicated 1.9 ± 1.1, 2.1 ± 1.1 and 16.5 ± 1.9% loss of OHCs from apical, middle and basal turns, respectively. No significant loss of OHCs was observed in the apical and middle turns of the cochlea in caffeine + cisplatin treatment group, in spite of distinct hearing loss observed in this group at lower to middle frequencies. In the basal turn, caffeine pre-treatment decreased cisplatin-induced loss of OHCs to 9.1 ± 1.3%, which was significantly less than that induced by cisplatin alone in the same region of the cochlea (Fig. 2B). This disparity between the increased ABR threshold (Fig. 1B) and reduced loss of OHC in caffeine + cisplatin group indicates that the additional hearing loss experienced by this treatment group is not due to the loss of OHCs. Trans-tympanic administration of *R*-PIA was able to reduce the loss of OHCs produced by caffeine + cisplatin in the basal turn (3.0 ± 1.3%), which underscores the otoprotective action of A_1_AR against OHC damage. Administration of vehicle, caffeine or *R*-PIA alone did not show any significant loss of OHCs in all the three turns of the cochlea.

### Caffeine exacerbates cisplatin-induced cochlear synaptopathy in rats

**Figure 3:**
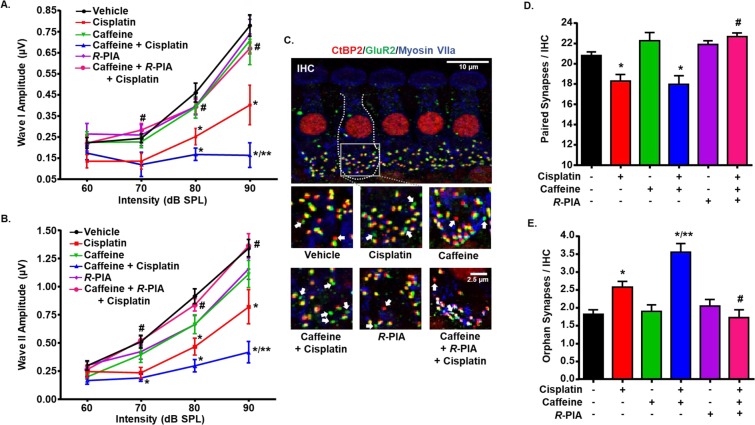
Caffeine pre-treatment enhances cisplatin-induced cochlear synaptopathy in rats. Amplitudes of ABR wave I (**A**) and wave II (**B**) of different treatment groups at 60, 70, 80, and 90 dB SPL (32 kHz) was computed from sorted ABR wave forms. Cisplatin significantly decreased supra-threshold wave I and II amplitudes, indicative of cochlear synaptopathy. Caffeine pre-treatment further reduced wave I and II amplitudes, suggesting worsening of cisplatin-induced hearing loss. The data is plotted as mean ± SEM (n ≥ 6). Asterisks, *p < 0.05 *vs*. vehicle; **p < 0.05 *vs*. cisplatin; and ^#^*p* < 0.05 *vs*. caffeine + cisplatin (two-way ANOVA). (**C**) Micro-dissected whole mount sections were stained with hair cell marker, myosin VIIa (blue), pre-synaptic marker, CtBP2 (red), and post-synaptic marker, GluR2 (green). Representative images from the basal turn shows ribbon synapses represented by co-localization of CtBP2 and GluR2. Orphan synapses were stained with either CtBP2 or GluR2 alone (indicated by white arrows). Total number of ribbon synapses and orphan synapses per IHC were calculated and plotted in (**D**,**E**), respectively. Caffeine pre-treatment did not alter cisplatin-induced decrease of paired ribbon synapses. However, caffeine significantly increased the number of orphan synapses as compared to cisplatin, which is then blocked by *R*-PIA. Major scale bar: 10 µm, minor scale bar: 2.5 µm. Data in (**D**,**E**) indicate mean ± SEM (n ≥ 4). Asterisks, *p < 0.05 *vs*. vehicle, **p < 0.05 *vs*. cisplatin and ^#^*p* < 0.05 *vs*. caffeine + cisplatin (one-way ANOVA).

In order to address the discrepancy between the enhanced ABR threshold shift which was not coupled to greater OHC loss in the basal turn, we examined the degree of induction of cochlear synaptopathy by cisplatin and caffeine + cisplatin treatment groups. Cochlear synaptopathy is the degeneration of auditory nerve fibers usually observed after noise exposure^25^ and aging^26^. Cochlear synaptopathy is characterized by the loss of synapses between IHCs and type I afferent auditory nerve fibers connecting the SGNs which results in signal coding deficits and is reflected in the reduction of supra-threshold ABR wave I amplitude^27^. We therefore analyzed ABR wave I amplitude in response to different sound intensities (60, 70, 80 and 90 dB SPL) at 32 kHz, which is the most sensitive frequency of the auditory spectrum in rats. As expected, the average wave I amplitude elicited in vehicle treated animals was significantly reduced following cisplatin treatment at 80 dB (*p* = 0.034) and 90 dB (*p* < 0.0001) (Fig. 3A). The decrease in wave I amplitude by cisplatin at 90 dB was further reduced by pre-treatment with caffeine (*p* = 0.016 vs. cisplatin). We also analyzed supra-threshold ABR wave II amplitude which represents the activity of cochlear nucleus^28^. Similar to wave I amplitude, supra-threshold wave II amplitude was significantly decreased by cisplatin (80 dB: *p* = 0.0003 and 90 dB: *p* < 0.0001 vs. vehicle), which was further diminished by caffeine co-administration (90 dB: *p* = 0.013 vs. cisplatin) (Fig. 3B). These decreases in wave I and wave II amplitudes were attenuated in rats pre-treated with trans-tympanic *R*-PIA (70 dB: *p* < 0.05, 80 dB: *p* < 0.005 and 90 dB: *p* < 0.0001 vs. caffeine + cisplatin), implicating a protective role of the A_1_AR at these different targets along the auditory pathway. Overall, these data suggest that reductions in wave I amplitudes could contribute to the additional hearing loss produced by caffeine in the cisplatin-treated rats.

Reductions in ABR wave I and II amplitudes produced by cisplatin are likely associated with the loss of ribbon synapses between IHCs and auditory nerve terminals^29^. Loss of ribbon synapses has been reported following exposure to loud noise^25^, treatment with ototoxic drugs, such as cisplatin^30,31^ and aminoglycosides^32^, and aging^26^. A functional afferent synapse includes presynaptic ribbon, the main component of which is the RIBEYE protein, and postsynaptic active zone containing glutamate receptors, such as GluR2. RIBEYE ribbon protein can be labeled by antibodies against C-terminal binding protein 2 (CtBP2). The loss of afferent synapses were assessed by immunolabeling of rat whole-mount sections with antibodies against CtBP2 and GluR2, while myosin VIIa was used to label IHCs. The average number of paired synaptic ribbons (showing both CtBP2 and GluR2 staining) per IHC in the basal turn of vehicle treated rats was 20.8 ± 0.4, which was significantly reduced to 18.3 ± 0.6 following cisplatin treatment (Fig. 3C,D). The number of orphan synapses also increased from 1.8 ± 0.1 in vehicle treated rats to 2.6 ± 0.2 per IHC by cisplatin (Fig. 3E). Pre-treatment with caffeine did not significantly reduce the number of paired synapses (17.9 ± 0.8 per IHC) more than that observed with cisplatin treatment group alone. In contrast, the number of orphan synapses were significantly increased to 3.6 ± 0.2 in caffeine + cisplatin group as compared to cisplatin alone (*p* = 0.005). These findings suggest that loss of paired ribbon synapses alone could not account for the additional hearing loss produced in the caffeine + cisplatin. Other factors, including differential deficits in synaptic functions, differential loss of SGNs or differences in the generation of inflammatory and oxidative stress mechanisms (see below) could possibly contribute. Pre-treatment with *R*-PIA by trans-tympanic injection significantly inhibited both caffeine + cisplatin-mediated loss of paired synapses and the increase in the number of orphan synapses (Fig. 3D,E), suggesting the overall efficacy of *R*-PIA in preserving synaptic integrity, at least in the basal turn of the cochlea.

**Figure 4:**
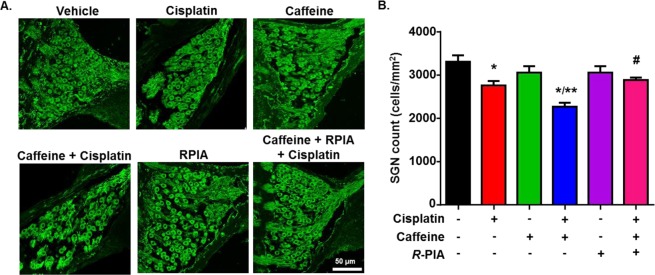
Pre-treatment with caffeine exacerbates cisplatin-induced SGN loss. (**A**) Mid-modiolar sections of the cochlea from treated animals were immunolabelled with neurofilament H antibody to stain SGN (green). Representative images from the basal turn of the cochlea are shown. Scale bar: 50 μm. (**B**) Bar graph plotted from the data presented in (**A**) shows that caffeine pre-treatment significantly decreased SGN count more than cisplatin alone, which is then blocked by *R*-PIA. Data indicate mean ± SEM (n ≥ 4). *p < 0.05 *vs*. vehicle, **p < 0.05 *vs*. cisplatin and ^#^*p* < 0.05 *vs*. caffeine + cisplatin (one-way ANOVA).

The degeneration of SGNs in response to an ototoxic insult is a secondary event to the loss of hair cells^25,33,34^ and could thus explain the additional hearing loss produced by caffeine + cisplatin. We evaluated the survival of SGNs by counting the number of SGN cell bodies within Rosenthal’s canal. Cisplatin treatment reduced the number of SGNs by 16.5 ± 3.1% (*p* = 0.009) as compared to vehicle treated group (Fig. 4A,B). Caffeine + cisplatin group demonstrated a 31.4 ± 2.8% decrease in SGN count, which was significantly greater than what was observed with cisplatin alone (*p* = 0.01). Pre-treatment with *R*-PIA inhibited the caffeine + cisplatin-induced loss of SGN (12.8 ± 1.7, *p* = 0.001), which is indicative of the cytoprotective effect of A_1_AR. These findings suggest that loss of ribbon synapses in addition to the degeneration of SGN could account for the additional hearing loss caused by caffeine + cisplatin treatments.

### Caffeine enhances cisplatin-mediated inflammatory response in rat cochlea

**Figure 5:**
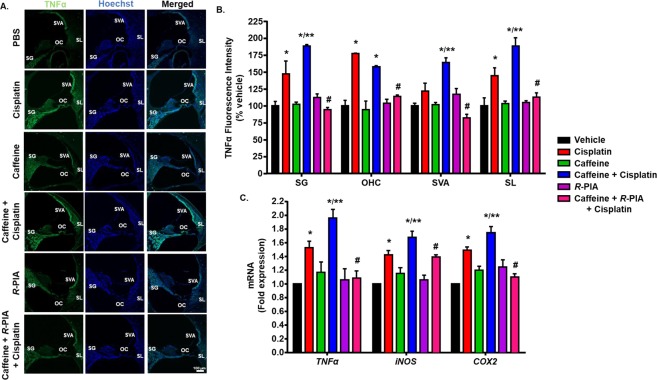
Caffeine enhances cisplatin–induced expression of inflammatory genes in the rat cochlea. (**A**) Cochleae from treated animals were collected and processed for mid-modiolar sections. These sections were immunolabelled with TNFα (green) antibody along with Hoechst (blue) to stain the cell nuclei. Representative images from the basal turn of the cochlea are shown. Scale bar represents 100 μm. (**B**) Bar graph plotted from the data presented in (**A**) show that cisplatin increased TNFα immunoreactivity in spiral ganglion (SG), OHC in the organ of Corti (OC) and spiral ligament (SL) but not in stria vascularis (SVA). Caffeine enhanced the increases by cisplatin in all regions of the cochlea except OHC. Increases in TNFα immunoreactivity by caffeine + cisplatin was blocked by *R*-PIA. Data represents mean ± SEM (n ≥ 3). (**C**) Cochleae from treated animals were also used for real-time RT-PCR to determine the levels of *TNFα*, *iNOS* and *COX2*. Caffeine significantly increases the expression of these inflammatory genes more than cisplatin, which is then blocked by *R*-PIA. Data indicate fold differences in the mRNA levels ± SEM (n ≥ 3). Asterisks, *p < 0.05 *vs*. vehicle, **p < 0.05 *vs*. cisplatin and ^#^*p* < 0.05 *vs*. caffeine + cisplatin (one-way ANOVA).

Previous studies have reported cochlear inflammation as one of the major contributors of cisplatin-induced hearing loss^12,13,35^. Activation of STAT1, a transcription factor, has been implicated in initiating an inflammatory process in the cochlea following cisplatin treatment^10,12^. STAT1 regulates the transcription of various pro-inflammatory cytokines, such as TNF-α^14^, iNOS^15^ and COX-2^16^. The up-regulation of these pro-inflammatory cytokines has been associated with cisplatin ototoxicity^12^. We recently reported that inhibition of STAT1 and its target genes by trans-tympanic administration of the A_1_AR agonist, *R*-PIA, protected from cisplatin-induced hearing loss by suppressing the inflammatory response^10^. Thus, inhibition of A_1_AR by caffeine could attenuate its otoprotective effect and intensify cisplatin ototoxicity by increasing the expression of inflammatory cytokines. To determine whether caffeine administration worsens cisplatin-induced inflammation, we examined the levels of TNF-α in mid-modiolar sections of the cochlea by immunohistochemistry. High TNF-α immunolabeling was observed 3 days following cisplatin administration in the SG, OHC and SL but not in SVA, compared with vehicle-treated rats (Fig. 5A,B). Caffeine treatment further increased cisplatin-induced TNF-α immunofluorescence in SG, SVA and SL but not in OHC. Trans-tympanic administration of *R*-PIA suppressed the increase in the immunolabeling of TNF-α by caffeine + cisplatin in all the regions of the cochlea.

We also investigated the expression of STAT1-regulated inflammatory genes, such as *TNF-α*, *COX-2* and *iNOS*, in whole cochlear RNA preparations. Cisplatin significantly increased the mRNA levels of *TNF-α*, *COX-2* and *iNOS* by 1.53 ± 0.1, 1.5 ± 0.04 and 1.4 ± 0.06 folds, respectively. Pre-treatment with caffeine further augmented cisplatin-induced mRNA levels of *TNF-α*, *COX-2* and *iNOS* to 1.96 ± 0.1, 1.75 ± 0.09 and 1.68 ± 0.09 folds, respectively. The expression of these genes were significantly reduced in rats pre-treated with *R*-PIA, indicating the functional role of A_1_AR in regulating the expression of these inflammatory genes in the cochlea (Fig. 5C). Accordingly, the hearing loss produced by caffeine + cisplatin can be attributed, at least in part, to the induction of inflammation in the cochlear cells.

### Caffeine treatment aggravates cisplatin-induced cytotoxicity in *in vitro* models

**Figure 6:**
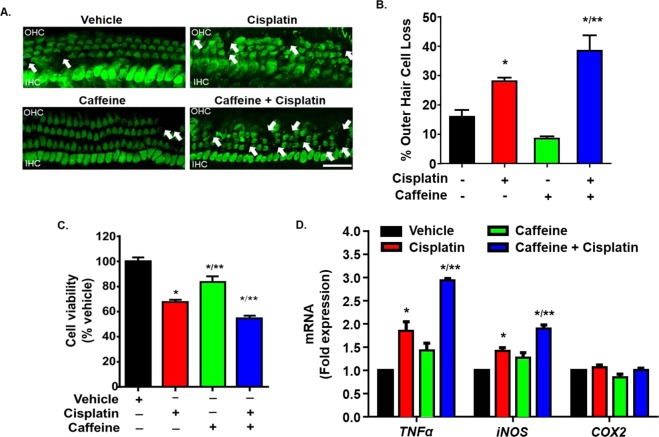
Caffeine exacerbates cisplatin-induced cell death in mouse explant cultures and UB/OC-1 cells. (**A**) Cochlear explants from neonatal (P3–P5) mice treated with cisplatin (20 µM) for 48 h show significant loss of myosin VIIa-stained OHCs (white arrows). The loss was enhanced by pre-treatment with caffeine (100 µM). Representative images are shown. Scale bar represents 25 µm. Data in (**B**) indicate mean percentage loss of OHCs ± SEM and were derived from (**A**) (n ≥ 3). (**C**) UB/OC-1 cells were treated with caffeine (100 μM) for 0.5 h, followed by cisplatin (20 μM) for an additional 24 h. Cell viability as determined by MTS assay showed that caffeine reduced the cell viability of cisplatin treated UB/OC-1 cells. Percent cell viability is presented as mean ± SEM of three independent experiments. (**D**) RNA was isolated using TRI reagent and expression of *TNF-α*, *iNOS* and *COX2* were determined by real time RT-PCR and normalized to *GAPDH*, a house keeping gene. Caffeine treatment enhanced the expression of inflammatory genes in cisplatin-treated UB/OC-1 cells. Data are presented as mean fold change ± SEM (n ≥ 3). Asterisks, *p < 0.05 *vs*. vehicle, **p < 0.05 *vs*. cisplatin (one-way ANOVA).

Caffeine and cisplatin-induced cytotoxicity was tested in cochlear organotypic cultures from postnatal days 3–5 (P3–P5) C57BL/6 mice. Cochlear explants are widely accepted substitute for *in vivo* cochlear models. We observed that cisplatin (20 μM) treatment for 48 hr significantly increased the loss of myosin VIIa-stained OHCs in the basal turn to 28 ± 1.2% from 15.8 ± 2.4% in vehicle-treated explant cultures (Fig. 6A,B). Cisplatin-induced loss of OHCs was exacerbated when the explant cultures were pre-treated with 100 μM caffeine (38.4 ± 5.3%). Treatment with caffeine alone did not show any significant change in the OHC loss as compared to vehicle-treated cultures. These data further underscore a tonic cytoprotective role of the A_1_AR which is disrupted by concurrent caffeine exposure.

We also checked the cytotoxic potential of caffeine in combination with cisplatin in UB/OC-1 cells, which is a mouse derived organ of Corti cell line. The UB/OC-1 cells were pre-treated with caffeine (100 μM) for 30 min prior to cisplatin (20 μM) administration and cell viability was determined 24 hr later using MTS assay. Cisplatin decreased cell viability of UB/OC-1 cells by ~33%. Caffeine alone reduced the cell viability by ~13% which was significant, but less than that of cisplatin. However, the combination of caffeine and cisplatin produced an additive effect, reducing cell viability by ~46% (Fig. 6C). The expression of inflammatory genes were also determined in UB/OC-1 cells. Cisplatin significantly increased the expression of *iNOS* and *TNF-α* but not *COX2*. Pre-treatment with caffeine further increased cisplatin-induced expression of *iNOS* and *TNF-α*. Caffeine treatment alone did not show any change in the expression of these inflammatory genes (Fig. 6D). These data suggest that caffeine could potentially exacerbate cisplatin ototoxicity *in vivo*. It is important to note that the UB/OC-1 cells were cultured in the presence of streptomycin, an aminoglycoside antibiotic, which may have synergistic effect with cisplatin in reducing cell viability. The data obtained here is not necessarily compromised as all the groups were cultured in the same amount of streptomycin (~21 μm) present in their media.

### Repeated caffeine administration enhances cisplatin-induced ototoxicity

**Figure 7:**
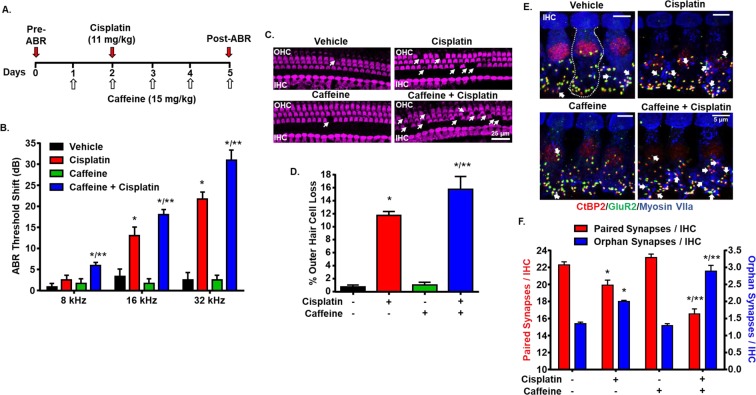
Repeated administration of caffeine enhances cisplatin-induced hearing loss, loss of OHCs and cochlear synaptopathy. (**A**) The timeline showing the experimental protocol for oral daily caffeine (15 mg/kg) administration. A single dose of cisplatin (11 mg/kg) was administered intraperitoneally a day after the first caffeine dose. Pre-treatment ABR was measured a day before caffeine was administered and post-treatment ABR was recorded 72 h following cisplatin administration and after the final caffeine treatment. (**B**) Daily administration of caffeine potentiated cisplatin-induced hearing loss at all the frequencies tested. Data indicate mean ± SEM of five rats. (**C**) Cochlear whole-mount sections stained with Myosin VIIa (magenta) show significant OHC loss (white arrows) in the base by cisplatin. Treatment with caffeine further increased cisplatin-induced OHC loss. Scale bar represents 25 μm. (**D**) Graphical representation of data presented in (**C**) showing percentage of OHC loss in the basal turn. Approximately 150 OHCs were counted per cochlea. Data indicates mean ± SEM (n ≥ 3). (**E**) Whole-mount sections from the basal turn were stained with hair cell marker, myosin VIIa (blue), pre-synaptic marker, CtBP2 (red), and post-synaptic marker, GluR2 (green). Co-localization of CtBP2 and GluR2 represents ribbon synapses, whereas orphan synapses are either CtBP2 or GluR2 alone (white arrows). Scale bar represents 5 µm. Total number of ribbon synapses and orphan synapses per 15 IHCs/cochlea were counted manually and plotted in (**F**). Repeated administration of caffeine enhanced cisplatin-induced synaptopathy by reducing the number of ribbon synapses and increasing the number of orphan synapses. Data indicate mean ± SEM (n ≥ 3). Asterisks, *p < 0.05 *vs*. vehicle and **p < 0.05 *vs*. cisplatin (one-way ANOVA).

Caffeine is rapidly and completely absorbed from the gastrointestinal tract within 45 min after oral administration^36^. It is quickly metabolized in liver, thereby limiting the amount that reaches the cochlea. Because of its short plasma half-life, multiple doses of caffeine is required to achieve a relatively constant concentration in the cochlea in order to attain a sustained blockade of the A_1_AR. To achieve this, we administered caffeine (15 mg/kg) to Wistar rats daily for five days via oral gavage. A single dose of cisplatin (11 mg/kg) was administered intraperitoneally a day after the first caffeine treatment. Post-treatment ABR was recorded 72 h following cisplatin administration and after the final caffeine treatment (Fig. 7A). The ABR thresholds were significantly elevated in cisplatin-treated rats, averaging 13 ± 2 and 21.7 ± 1.7 at 16 and 32 kHz, respectively (Fig. 7B). Cisplatin treatment did not produce a significant threshold shift at 8 kHz. Daily oral administration of caffeine in cisplatin-treated animals further elevated the ABR threshold which was significantly greater than cisplatin alone at all the frequencies tested. ABR threshold shifts in caffeine + cisplatin group were 5.8 ± 0.8, 18.0 ± 1.2 and 30.8 ± 2.4 at 8, 16 and 32 kHz, respectively. Daily administration of caffeine also aggravated cisplatin-induced damage to OHCs and cochlear synaptopathy in the basal turn of the cochlea. Cisplatin produced 11.8 ± 0.6% OHC damage in the basal turn which was increased to 15.7 ± 2% by repeated caffeine administration (Fig. 7C,D). Animals treated with vehicle or caffeine alone did not show any significant loss of OHCs in the basal turn. We also tested these whole-mount preparations for cochlear synaptopathy and observed that the average number of paired synaptic ribbons per IHC in vehicle treated animals was 22.3 ± 0.4, which was significantly reduced to 19.9 ± 0.6 following cisplatin treatment (Fig. 7E,F). Daily caffeine administration further reduced cisplatin-mediated loss of paired synapses to 16.5 ± 0.6 per IHC. Similarly, daily administered caffeine significantly increased the number of orphan synapses than cisplatin alone. Cisplatin treatment increased the number of orphan synapses from 1.3 ± 0.1 in vehicle treated rats to 2.0 ± 0.1 per IHC. Cisplatin-induced increase in the number of orphan synapses per IHC was further increased significantly to 2.9 ± 0.2 when these rats were treated daily with caffeine. These findings suggest that multiple doses of caffeine produce greater potentiation of cisplatin-induced ototoxicity than a single dose. In contrast, multiple oral daily doses of caffeine alone did not produce any ototoxic side effects.

## Discussion

The current study demonstrates exacerbation of cisplatin-induced hearing loss which results from concurrent consumption of caffeine. We demonstrate worsening of hearing loss following a single-dose administration of caffeine and even greater effects following daily applications of the drug for five days. Reversal of the negative effects of caffeine on cisplatin-induced hearing loss is observed by stimulating the A_1_AR with *R*-PIA, implicating this receptor as a target of caffeine an in otoprotection. These finding have significant implications giving the widespread consumption of caffeinated beverages worldwide and the use of cisplatin in various chemotherapeutic regimens for cancers.

Our data indirectly validate the role of endogenous adenosine and A_1_AR activation in protecting the cochlea against oxidative and inflammatory stressors, as observed with cisplatin. We and others have shown an otoprotective role of cochlear A_1_AR against cisplatin-induced hearing loss^10,19–21^. Furthermore, the A_1_AR protects against noise-induced hearing loss^37^ which resulted from loss of OHCs, SGNs and synaptic ribbons^29^. Protection was even observed when the agonist was administered 6 h following noise exposure^38^. In contrast, blockade of the A_1_AR by dipropylcyclopentylxanthine (DPCPX) reversed otoprotection provided by A_1_AR agonist^10,21^. One of the main mechanisms underlying the A_1_AR-dependent protection involves suppression of oxidative stress^10,19^ which reduced subsequent inflammatory processes in the cochlea and reduced cell apoptosis^10^. These actions have been shown to involve suppression of cisplatin-induced NOX3 NADPH oxidase function and expression and inhibition of the STAT1 transcription factor. These findings provide greater insight into the workings of the adenosine/A_1_AR signaling system which protects the cochlea against ototoxic drugs and acoustic trauma.

Based on its central role in cochlear defense, drugs which antagonize the A_1_AR would be expected to compromise hearing, especially in presence of stressors such as cisplatin. Coffee is widely consumed worldwide and contains relatively high levels of caffeine compared to other beverages. Lesser amounts of caffeine is consumed in tea, carbonated soft drinks, energy drinks and fruit-flavored drinks^39^. Moderate caffeine consumption (~400 mg/day) has been shown to provide a number of health benefits in healthy adults ranging from increased mental alertness, exercise endurance and weight loss to enabling better control of blood glucose^39^. However, our data would suggest that these levels of caffeine could contribute to hearing loss in humans with concurrent cisplatin chemotherapy. The dose of caffeine used in this study, 15 mg/kg per day, is equivalent to ~145 mg caffeine consumption per a 60 kg individual based on a previous publication on translation of doses used in animal studies to equivalent human doses^40^. This equivalent dose of caffeine is well under the 400 mg/day consumption level for a moderate coffee drinker. Furthermore, most coffee drinkers would habitually consume similar daily levels of caffeine over a prolonged period. Based on these findings one would anticipate that moderate levels of consumption of coffee could “sensitize” the cochlea to ototoxic drugs and/or acoustic trauma.

We show that increased hearing loss in animals treated with cisplatin, following consumption of a single dose of caffeine, was significant only at the high frequency (32 kHz) region of the cochlea. This region is more prone to cisplatin ototoxicity and our data suggest it is also more prone to sensitization by caffeine. Cisplatin ototoxicity was associated with increased loss of OHCs (but not IHCs) in the base of the cochlea, but this effect was not potentiated by caffeine. In fact, caffeine was able to provide some protection against OHC loss in the base. The reason for this is not clear but would implicate OHC dysfunction or targets other than hair cells in the auditory pathway which confer susceptiblity to caffeine. One likely target are the IHC ribbon synapses which were significantly decreased by cisplatin and even further decreased in the cisplatin plus caffeine group. Synapse loss and recovery are reflected by the amplitudes of wave I (measure of the function of ribbon synapses and auditory nerve) of the recorded ABRs. While cisplatin decreased wave I amplitudes, these effects were further reduced by caffeine. This effect is more obvious in Fig. 3A if one examines the rate of change in wave I amplitude versus stimulus intensity. There is a clear difference between the cisplatin versus the caffeine + cisplatin group. Thus, activation of the A_1_AR could play a dual role in reversing the synaptic loss produced by cisplatin alone and the combination of cisplatin and caffeine. A similar pattern of loss of wave II amplitude, which reflects activity of the cochlear nucleus, was observed between the cisplatin versus caffeine + cisplatin groups. Cisplatin suppressed wave II amplitude, which was further suppressed by caffeine, especially at high stimulus levels. Since these effects of caffeine on waves I and II were abolished by activation of the A_1_AR agonist, one could implicate this receptor subtype in protecting these auditory synapses. A recent study by Vlajkovic *et al*.^29^, using A_1_AR knockout mice in a noise-induced hearing loss paradigm, supports this conclusion. Interestingly, these investigators showed that A_2A_AR knockout mice were more protected against noise-induced hearing loss^29^ contrasting this receptor subtype with the A_1_AR.

The mechanism underlying synaptic loss and/or protection by the A_1_AR is not clear. In a previous study, we showed that the green tea extract, epigallocatechin gallate, protected against cisplatin-induced loss of IHC ribbon synapses^30^. Since epigallocatechin gallate possess antioxidant and anti-inflammatory properties, we speculated that these properties contribute to the preservation in ribbon synapses. Similarly, we have also shown that cannabinoid 2 (CB2) receptors present in the cochlea could protect these synapses^31^, possibly by targeting similar oxidative stress and inflammatory mechanisms. Noise-induced synaptopathy has been linked to presynaptic release of excessive glutamate^41^. The nerve fibers which are most susceptible possess low spontaneous firing rates and high thresholds^42^ and also express low levels of glutamate aspartate transporters (GLAST). Blockade of glutamate uptake leads to enhanced synaptic loss. Inhibition of presynaptic glutamate release by the A_1_AR^43^ could therefore serve as a mechanism of protection of the cochlea against cisplatin-induced synaptic loss. Caffeine, which serves as an antagonist of this receptor, would be expected to exacerbate the response to cisplatin. Noise-induced loss of ribbon synapses is associated with a loss of trophic support provided by neurotrophin-3 (NT-3), produced by IHC supporting cells^44^. This could reflect reductions in NT-3 production by these supporting cells or loss of supporting cells, resulting from increased oxidative and inflammatory stress in the vicinity of these cells. Administration of NT-3 and brain derived neurotrophic factors promotes regrowth of spiral ganglion nerve terminals and enable synapse regeneration *in vitro*^45^ and *in vivo*^46^. We speculate that oxidative^47^ and inflammatory stress^48^ produced by cisplatin could regulate NT-3 production in the cochlea. This action could be reversed by the A_1_AR which promotes both antioxidant and anti-inflammatory control^17^. An additional factor which could contribute to reductions in the number of paired synapses is the loss of SGNs, which contribute the postsynaptic component of the synapses. We showed that cisplatin and cisplatin + caffeine increased the loss of SGNs which were reduced by concurrent activation of the A_1_AR.

In summary, this study highlights a significant drug-drug interaction between cisplatin and caffeine *in vitro* and animal studies. Since caffeine is a commonly consumed drug in a number of beverages, such a negative interaction is likely in patients on a cisplatin-based chemotherapy regimen. Cisplatin has recently been shown to persist in the cochlea (SVA) and long bones for months and years following the last drug administration^49^. These long bones could serve as reservoirs of cisplatin in the body which contribute its prolonged toxicity^49^. As such, this drug interaction between caffeine and cisplatin could persist long after cisplatin administration has been terminated.

## Materials and Methods

### Drugs and reagents

All the drugs used in this study, including caffeine, N^6^-*R*-phenylisopropyladenosine (*R*-PIA) and cisplatin were purchase from Sigma-Aldrich. Different antibodies used in this study were purchased from different vendors and are as follows: anti-CtBP2 mouse IgG1 was obtained from BD Biosciences (#612044) while anti-glutamate receptor 2 (GluR2) mouse IgG2a was obtained from Millipore (#MAB397). Rabbit polyclonal myosin-VIIa was purchased from Proteus Biosciences (#25–6790) and TNFα goat polyclonal IgG was from Santa Cruz Biotechnology (sc-1348). Alexa Fluor^®^ 488 conjugated anti-neurofilament H antibody was purchased from Millipore (#MAB5256X). Secondary antibodies used were as follows: Alexa Fluor^TM^ 647 goat anti-mouse IgG1 (A-21240), Alexa Fluor^TM^ 488 goat anti-mouse IgG2a (A-21131) and Alexa Fluor^TM^ 647 donkey anti-goat IgG (A-21447) were obtained from Life Technologies. Rhodamine (TRITC) AffiniPure Donkey Anti-Rabbit IgG was purchased from Jackson ImmunoResearch Laboratories (711-025-152).

### Animal procedures

Adult male Wistar rats (weighing ~200 g) were purchased from Envigo and housed in the Division of Laboratory Animal Medicine (DLAM) facility of Southern Illinois University School of Medicine. The rats were housed in standard cages in a temperature-controlled environment with 12 h light/dark cycle with free access to food and water. They were anesthetized using a mixture of ketamine (90 mg/kg) and xylazine (17 mg/kg) given intraperitoneally and the depth of anesthesia was determined by the absence of reflex to toe pinch. In case the depth of anesthesia was determined to be insufficient, the rats were administered additional dose of the mixture. Pre-treatment auditory brainstem responses (ABRs) were performed on anesthetized rats in a sound proof chamber. Caffeine (15 mg/kg) was administered via oral gavage whereas *R*-PIA (1 μM) was delivered into the middle ear through trans-tympanic route. Cisplatin (11 mg/kg) was administered intraperitoneally and post-treatment ABRs were measured 72 h later. Following the ABR measurements, the rats were sacrificed by decapitation under anesthesia. One cochlea from each animal was fixed using cochlear perfusion of 4% paraformaldehyde in PBS for immunohistochemistry studies and the other cochlea was perfused with RNA*later* (Thermo Fisher Scientific) for RNA extraction. All animal procedures were approved by the Southern Illinois University School of Medicine Laboratory Animal Care and Use Committee (LACUC). All experimental procedures were performed in accordance with the animal protocol approved by the SIU School of Medicine LACUC.

### Auditory brainstem response

Auditory brainstem responses are sound-evoked electrophysiological auditory responses derived from the activity of the auditory nerve and central auditory pathways. The ABR were determined as described previously^50^. Briefly, anesthetized rats were placed inside a sound proof booth in the prone position on a heating pad maintained at 37 °C. Stainless steel electrodes were inserted subdermally in the hind flank muscle region (ground), between the two ears directly atop the skull (positive) and under the pinna of each ear (negative) to detect the ABR. Computer generated acoustic stimuli was applied as tone burst with 5 ms duration and 1 ms rise/fall time at frequencies 8, 16 and 32 kHz using high frequency transducers (Intelligent Hearing System) connected to a plastic tube placed into the external auditory canal of each ear. Animals were tested with a stimulus intensity series that was initiated at 90 dB sound pressure level (SPL) with 10 dB step and reached the minimum at 10 dB SPL. ABR waves II and III complex was used to determine ABR threshold for each frequency. The ABR threshold was defined as the lowest intensity capable of evoking a reproducible and visually distinguishable response of waves II/III complex. Threshold shift represents the difference in threshold measured after treatment (post-treatment ABR) compared with threshold obtained prior to treatment (pre-treatment ABR). The amplitudes (voltage measured from peak to trough) of waves I and II were determined at supra-threshold intensities (60, 70, 80 and 90 dB) from the ABRs obtained from the different treatment groups.

### Cochlear whole-mount preparation for OHC and ribbon synapse counts

The isolated rat cochleae were perfused with 4% paraformaldehyde in PBS through the oval and round window and were fixed in the same solution for 24 h at 4 °C. Cochleae were then decalcified in 120 mM EDTA (replaced daily) with constant stirring at room temperature for 3 weeks. From the decalcified cochleae the basilar membrane containing the organ of Corti was carefully microdissected and separated into three segments: apical, middle and basal turn, which corresponds to 8, 16 and 32 kHz frequencies, respectively. Microdissected cochlear whole-mount samples were labeled with antibodies against myosin VIIa, CtBP2, and GluR2 for staining of hair cells, presynaptic ribbon and post-synaptic glutamate receptors, respectively. The fluorescently labeled samples were imaged by Leica SP5 II scanning confocal microscope. In each sample, the number of missing OHCs were counted manually from ~150 OHC per turn and presented as percent loss of the total number of OHC. Ribbon synapses from the IHCs were imaged at 100X magnification to capture at least 15 IHCs per image. Paired (CtBP2 + GluR2 immunolabeling apposing each other) and orphan or unpaired (either CtBP2 or GluR2 missing) synaptic ribbons were counted manually from each image and presented as total number of synapses per IHC.

### SGN count

Decalcified cochleae were processed for cryosectioning as described previously^50^. Serial mid-modiolar sections (10 μm thickness) were immunolabelled with neurofilament H antibody using standard immunofluorescence method described below to stain the SGN. The sections were then imaged using Leica SP5 II scanning confocal microscope at 40X magnification. To count the number of SGN, neurofilament H labelled cell bodies within the Rosenthal’s canal in the basal turn were counted manually from maximum intensity projection images of each section. The number of SGN counted from sections were normalized to the cross-sectional area of Rosenthal’s canal and averaged and presented as SGN count/1 mm^2^.

### Immunohistochemistry

Immunostaining of cochlear whole-mount preparations, explant cultures and mid-modiolar sections (10 μm thickness) was initiated by methanol permeabilization step. For this the tissue sections were kept in ice-cold 100% methanol for 10 min at −20 °C and then rinsed once in 1X PBS for 5 min. Following this the tissue sections were blocked in blocking buffer (1X PBS + 5% normal donkey serum + 0.3% Triton X-100) for 2 hr at room temperature. The specimen were then incubated overnight at 4 °C in primary antibody prepared in antibody dilution buffer (1X PBS + 1% BSA + 0.3% Triton X-100), after which they were washed three times for 5 min with 1X PBS. They were then incubated in secondary antibodies prepared in antibody dilution buffer for 3 h in dark at room temperature and then washed three times for 5 min with 1X PBS. Sections were then stained with Hoechst in the dark for 20 min at room temperature and mounted on slides using ProLong^®^ Diamond Antifade Mountant (Invitrogen). The samples containing hair cells, ribbon synapses and SGN were imaged by Leica SP5 II scanning confocal microscope whereas samples immunolabeled with TNF-α were imaged by Zeiss LSM800 scanning confocal microscope. The TNF-α immunofluorescent intensity was measured using ZEN 2.5 (blue edition) software by Zeiss using sequential imaging for excitation wavelengths. Although not all the images were taken during the same imaging session, all the images had same laser illumination settings for all the groups (n ≥ 3 cochleae/group). First, the mean fluorescent intensity was obtained by selecting the region of interest (ROI) from 2 to 3 sections per cochlea. Next, the region next to the ROI was selected that had no fluorescence to get the background fluorescent value. This value was then deducted from the mean fluorescent intensity of ROI to compute the total fluorescent intensity.

### RNA isolation and real-time RT-PCR

Isolated cochleae were cleaned of the extraneous tissue and crushed in 1 ml of TRI reagent^®^ (Sigma-Aldrich) using a homogenizer. About 200 μl of 100% chloroform was added and the tube was shaken vigorously for 15 s and centrifuged at 12,000 g for 15 min. The top clear phase (~200 μl) containing RNA was collected carefully and transferred to another tube. RNA was extracted by adding 500 μl of ice-cold 100% isopropanol and mixing the tube by inverting it several times followed by centrifugation at 12,000 g for 15 min. The supernatant was discarded and the RNA pellet was washed once with ice-cold 100% ethanol and then twice with cold 75% ethanol. The ethanol was removed and the tube was air dried briefly. The RNA pellet was resuspended in nuclease-free water and the purity and concentration of RNA was determined by measuring optical density at 260, 280, and 230 nm by NanoDrop^®^ ND-1000 spectrophotometer. Total RNA from UB/OC-1 cells were also isolated as described above.

Total RNA (500 ng) was reverse transcribed in 20 μl reactions using iScript Select cDNA Synthesis Kit (Bio-Rad) as per the manufacturers protocol. The reaction mixture was setup as follows: 500 ng of total RNA, 4 μl of 5X iScript select reaction mix, 2 μl of GSP enhancer solution, 1 μl of iScript reverse transcriptase and nuclease-free water to bring the total volume to 20 μl. The reaction mix was incubated at 42 °C for 60 min and 85 °C for 5 min. This cDNA reaction mix was used for RT-PCR which was setup as follows: 2 μl of cDNA, 0.5 μl of each primer (50 pM stock) and 10 μl of Fast SYBR Green Mastermix (Thermo Fisher Scientific), adjusted to a total volume of 20 μl with nuclease-free water. Cycling conditions were: 95 °C for 20 sec followed by 40 cycles at 95 °C for 3 sec and 60 °C for 30 s each. Amplification and detection was performed using StepOnePlus Real-Time PCR System (Thermo Fisher Scientific). The mRNA expression were normalized to the levels of GAPDH. The cycle threshold (*C*t) was determined by the number of cycles at which the samples reached threshold fluorescent intensity. The relative change in mRNA levels of a specific gene between the vehicle and treated group was quantified by the following formula: 2^−ΔΔCt^. The nucleotide sequences of rodent primer sets were based on homologous sequences of rat and mouse cDNA sequence. The primers were purchased from Sigma-Aldrich and the primer sets used were: Rodent-TNF-α (sense): 5′-CAGACCCTCACACTCAGATCA-3′; Rodent-TNF-α (antisense): 5′-TGAAGAGAACCTGGGAGTAGA-3′; Rodent-COX-2 (sense): 5′-TGATCGAAGACTACGTGCAAC-3′; Rodent-COX-2 (antisense): 5′-GTACTCCTGGTCTTCAATGTT-3′; Rodent-iNOS (sense): 5′-CATTCTACTACTACCAGATC-3′; Rodent-iNOS (antisense): 5′- ATGTGCTTGTAACCACCAG-3′ and Rodent-GAPDH (sense): 5′-ATGGTGAAGGTCGGTGTGAAC-3′; Rodent-GAPDH (antisense): 5-TGTAGTTGAGGTCAATGAAGG-3′;

### Neonatal murine cochlear explant dissection and culture

Postnatal days 3–5 (P3–P5) C57BL/6 mice pups were anesthetized using isoflurane and then decapitated. Using a microscope, the temporal bone was removed from the skull and cochlea was located and isolated in cold dissection media (1X Hank’s balanced salt solution (Gibco)). The lateral wall was dissected away and the whole basilar membrane containing the organ of Corti was isolated and transferred to a 35 mm tissue culture dish with a coverslip pre-coated with poly-l-ornithine and laminin. The cochlear explants were cultured in DMEM with 1% FBS and 50 µg/ml ampicillin at 37 °C in 5% CO_2_ for 1 day. These explant cultures were then pre-treated with either vehicle or caffeine (100 µM) for 30 min followed by cisplatin (20 µM) for 48 hr. After the treatment end point, the cultures were fixed in 4% paraformaldehyde and stained with myosin VIIa to determine the OHC loss. One random image of 150 µm was captured from the basal turn using Leica SP5 II scanning confocal microscope, and the number of missing OHCs were counted manually. At least three independent cochlea were tested for each treatment.

### Cell culture

Immortalized mouse organ of Corti cells, UB/OC-1, were kindly provided by Dr. Matthew Holley (The University of Sheffield, UK). These cells were cultured in RPMI-1640 media (HyClone) supplemented with 10% FetalClone^®^ II serum (Hyclone), 5% penicillin–streptomycin (Invitrogen) and normocin (InvivoGen). UB/OC-1 cells were kept at 33 °C in a humidified incubator with 10% CO_2_. Cells were cultured thrice a week for passaging and sub-confluent monolayer of cells were used for experiments.

### Cell viability (MTS) assay

*In vitro* cell viability of UB/OC-1 cells was tested using CellTiter 96^®^ AQueous One Solution Cell Proliferation Assay Kit (Promega). UB/OC-1 cells (2500 cells per well) were seeded into a 96-well plate. After respective treatments, cells were allowed to grow for 24 h, following which 20 µl of CellTiter 96 AQueous One Solution reagent was added to each well in 100 µl of total volume of media. Cells were incubated for 3 to 4 h in 33 °C incubator and absorbance was recorded at 490 nm using an ELISA plate reader. The absorbance is directly proportional to the number of living cells and is expressed as a percent relative to vehicle-treated cells. The experiment was repeated at least three times and average absorbance from independent repeats were used for statistical analyses.

### Statistics

Data are presented as mean ± standard error of mean (SEM). Statistical significance of differences among groups were tested using one-way analysis of variance (ANOVA) unless specified, followed by Bonferroni’s multiple comparison’s test using GraphPad Prism version 6.07 for Windows. *P* value < 0.05 was considered significant.

## Data Availability

The authors confirm that the data supporting the findings of this study are available within the article.

## References

[1] Schmitt NC, Rubel EW, Nathanson NM (2009). Cisplatin-induced hair cell death requires STAT1 and is attenuated by epigallocatechin gallate. The Journal of neuroscience: the official journal of the Society for Neuroscience.

[2] Sheth S, Mukherjea D, Rybak LP, Ramkumar V (2017). Mechanisms of Cisplatin-Induced Ototoxicity and Otoprotection. Frontiers in cellular neuroscience.

[3] Jadali A, Ying YM, Kwan KY (2017). Activation of CHK1 in Supporting Cells Indirectly Promotes Hair Cell Survival. Frontiers in cellular neuroscience.

[4] Lee JE (2004). Mechanisms of apoptosis induced by cisplatin in marginal cells in mouse stria vascularis. ORL; journal for oto-rhino-laryngology and its related specialties.

[5] Meech RP, Campbell KC, Hughes LP, Rybak LP (1998). A semiquantitative analysis of the effects of cisplatin on the rat stria vascularis. Hearing research.

[6] van Ruijven MW, de Groot JC, Hendriksen F, Smoorenburg GF (2005). Immunohistochemical detection of platinated DNA in the cochlea of cisplatin-treated guinea pigs. Hearing research.

[7] Liu Wenwen, Xu Xiaochen, Fan Zhaomin, Sun Gaoying, Han Yuechen, Zhang Daogong, Xu Lei, Wang Mingming, Wang Xue, Zhang Shasha, Tang Mingliang, Li Jianfeng, Chai Renjie, Wang Haibo (2019). Wnt Signaling Activates TP53-Induced Glycolysis and Apoptosis Regulator and Protects Against Cisplatin-Induced Spiral Ganglion Neuron Damage in the Mouse Cochlea. Antioxidants & Redox Signaling.

[8] Wu X (2017). Allicin protects auditory hair cells and spiral ganglion neurons from cisplatin - Induced apoptosis. Neuropharmacology.

[9] Zheng JL, Stewart RR, Gao WQ (1995). Neurotrophin-4/5 enhances survival of cultured spiral ganglion neurons and protects them from cisplatin neurotoxicity. The Journal of neuroscience: the official journal of the Society for Neuroscience.

[10] Kaur T (2016). Adenosine A1 Receptor Protects Against Cisplatin Ototoxicity by Suppressing the NOX3/STAT1 Inflammatory Pathway in the Cochlea. The Journal of neuroscience: the official journal of the Society for Neuroscience.

[11] Nakai Y (1982). Ototoxicity of the anticancer drug cisplatin. An experimental study. Acta oto-laryngologica.

[12] Kaur T (2011). Short interfering RNA against STAT1 attenuates cisplatin-induced ototoxicity in the rat by suppressing inflammation. Cell death & disease.

[13] Mukherjea D (2011). NOX3 NADPH oxidase couples transient receptor potential vanilloid 1 to signal transducer and activator of transcription 1-mediated inflammation and hearing loss. Antioxidants & redox signaling.

[14] Sugawara I, Yamada H, Mizuno S (2004). STAT1 knockout mice are highly susceptible to pulmonary mycobacterial infection. The Tohoku journal of experimental medicine.

[15] Jacobs AT, Ignarro LJ (2001). Lipopolysaccharide-induced expression of interferon-beta mediates the timing of inducible nitric-oxide synthase induction in RAW 264.7 macrophages. The Journal of biological chemistry.

[16] Xuan YT (2003). Mechanism of cyclooxygenase-2 upregulation in late preconditioning. Journal of molecular and cellular cardiology.

[17] Sheth S, Brito R, Mukherjea D, Rybak LP, Ramkumar V (2014). Adenosine receptors: expression, function and regulation. International journal of molecular sciences.

[18] Vlajkovic SM, Abi S, Wang CJ, Housley GD, Thorne PR (2007). Differential distribution of adenosine receptors in rat cochlea. Cell and tissue research.

[19] Ford MS, Maggirwar SB, Rybak LP, Whitworth C, Ramkumar V (1997). Expression and function of adenosine receptors in the chinchilla cochlea. Hearing research.

[20] Gunewardene N, Guo CX, Wong ACY, Thorne PR, Vlajkovic SM (2013). Adenosine amine congener ameliorates cisplatin-induced hearing loss. World Journal of Otorhinolaryngology.

[21] Whitworth CA, Ramkumar V, Jones B, Tsukasaki N, Rybak LP (2004). Protection against cisplatin ototoxicity by adenosine agonists. Biochemical pharmacology.

[22] Heckman MA, Weil J, Gonzalez de Mejia E (2010). Caffeine (1, 3, 7-trimethylxanthine) in foods: a comprehensive review on consumption, functionality, safety, and regulatory matters. Journal of food science.

[23] Ferre S (2008). An update on the mechanisms of the psychostimulant effects of caffeine. Journal of neurochemistry.

[24] Mujica-Mota MA, Gasbarrino K, Rappaport JM, Shapiro RS, Daniel SJ (2014). The effect of caffeine on hearing in a guinea pig model of acoustic trauma. American journal of otolaryngology.

[25] Kujawa SG, Liberman MC (2009). Adding insult to injury: cochlear nerve degeneration after “temporary” noise-induced hearing loss. The Journal of neuroscience: the official journal of the Society for Neuroscience.

[26] Sergeyenko Y, Lall K, Liberman MC, Kujawa SG (2013). Age-related cochlear synaptopathy: an early-onset contributor to auditory functional decline. The Journal of neuroscience: the official journal of the Society for Neuroscience.

[27] Kobel M, Le Prell CG, Liu J, Hawks JW, Bao J (2017). Noise-induced cochlear synaptopathy: Past findings and future studies. Hearing research.

[28] Tait C, Miller J, Cycowicz Y, Sohmer H (1987). Experimental analyses of the source of ABR wave II. Archives of oto-rhino-laryngology.

[29] Vlajkovic SM (2017). Adenosine receptors regulate susceptibility to noise-induced neural injury in the mouse cochlea and hearing loss. Hearing research.

[30] Borse V (2017). Epigallocatechin-3-gallate, a prototypic chemopreventative agent for protection against cisplatin-based ototoxicity. Cell death & disease.

[31] Ghosh S (2018). The Endocannabinoid/Cannabinoid Receptor 2 System Protects Against Cisplatin-Induced Hearing Loss. Frontiers in cellular neuroscience.

[32] Liu K (2013). Cochlear inner hair cell ribbon synapse is the primary target of ototoxic aminoglycoside stimuli. Molecular neurobiology.

[33] Bae WY, Kim LS, Hur DY, Jeong SW, Kim JR (2008). Secondary apoptosis of spiral ganglion cells induced by aminoglycoside: Fas-Fas ligand signaling pathway. The Laryngoscope.

[34] McFadden SL, Ding D, Jiang H, Salvi RJ (2004). Time course of efferent fiber and spiral ganglion cell degeneration following complete hair cell loss in the chinchilla. Brain research.

[35] Kalinec GM, Lomberk G, Urrutia RA, Kalinec F (2017). Resolution of Cochlear Inflammation: Novel Target for Preventing or Ameliorating Drug-, Noise- and Age-related Hearing Loss. Frontiers in cellular neuroscience.

[36] Fredholm BB, Battig K, Holmen J, Nehlig A, Zvartau EE (1999). Actions of caffeine in the brain with special reference to factors that contribute to its widespread use. Pharmacological reviews.

[37] Hu BH, Zheng XY, McFadden SL, Kopke RD, Henderson D (1997). R-phenylisopropyladenosine attenuates noise-induced hearing loss in the chinchilla. Hearing research.

[38] Wong AC (2010). Post exposure administration of A(1) adenosine receptor agonists attenuates noise-induced hearing loss. Hearing research.

[39] Mitchell DC, Knight CA, Hockenberry J, Teplansky R, Hartman TJ (2014). Beverage caffeine intakes in the U.S. Food and chemical toxicology: an international journal published for the British Industrial Biological Research Association.

[40] Reagan-Shaw S, Nihal M, Ahmad N (2008). Dose translation from animal to human studies revisited. FASEB journal: official publication of the Federation of American Societies for Experimental Biology.

[41] Pujol R, Puel JL, Gervais d’Aldin C, Eybalin M (1993). Pathophysiology of the glutamatergic synapses in the cochlea. Acta oto-laryngologica.

[42] Furman AC, Kujawa SG, Liberman MC (2013). Noise-induced cochlear neuropathy is selective for fibers with low spontaneous rates. Journal of neurophysiology.

[43] Morelli M, Carta AR, Kachroo A, Schwarzschild MA (2010). Pathophysiological roles for purines: adenosine, caffeine and urate. Progress in brain research.

[44] Green SH, Bailey E, Wang Q, Davis RL (2012). The Trk A, B, C’s of neurotrophins in the cochlea. Anatomical record.

[45] Wang Q, Green SH (2011). Functional role of neurotrophin-3 in synapse regeneration by spiral ganglion neurons on inner hair cells after excitotoxic trauma *in vitro*. The Journal of neuroscience: the official journal of the Society for Neuroscience.

[46] Wan, G., Gomez-Casati, M. E., Gigliello, A. R., Liberman, M. C. & Corfas, G. Neurotrophin-3 regulates ribbon synapse density in the cochlea and induces synapse regeneration after acoustic trauma. *eLife* **3**, 10.7554/eLife.03564 (2014). 10.7554/eLife.03564 PMC4227045 25329343

[47] Blossom SJ, Melnyk S, Cooney CA, Gilbert KM, James SJ (2012). Postnatal exposure to trichloroethylene alters glutathione redox homeostasis, methylation potential, and neurotrophin expression in the mouse hippocampus. Neurotoxicology.

[48] Fuentes-Santamaria V (2014). Glia-related mechanisms in the anteroventral cochlear nucleus of the adult rat in response to unilateral conductive hearing loss. Frontiers in neuroscience.

[49] Breglio AM (2017). Cisplatin is retained in the cochlea indefinitely following chemotherapy. Nature communications.

[50] Sheehan, K., Sheth, S., Mukherjea, D., Rybak, L. P. & Ramkumar, V. Trans-Tympanic Drug Delivery for the Treatment of Ototoxicity. *Journal of visualized experiments: JoVE*, 10.3791/56564 (2018). 10.3791/56564 PMC5931769 29608150

